# Airway clearance treatments in bronchiectasis: feasibility of linking survey results to registry data and a survey of patients’ and physiotherapists’ practices

**DOI:** 10.1183/23120541.00540-2022

**Published:** 2023-03-20

**Authors:** Rebecca H. McLeese, Katherine O'Neill, Brenda O'Neill, James D. Chalmers, Jeanette Boyd, Anthony De Soyza, Ryan McChrystal, Megan L. Crichton, Judy M. Bradley

**Affiliations:** 1 Wellcome-Wolfson Institute for Experimental Medicine, Queen's University Belfast, Belfast, UK; 2 Centre for Health and Rehabilitation Technologies, Ulster University, Coleraine, UK; 3 Scottish Centre for Respiratory Research, University of Dundee, Ninewells Hospital and Medical School, Dundee, UK; 4 European Lung Foundation, Sheffield, UK; 5 Freeman Hospital Newcastle, Newcastle, UK; 6 These authors contributed equally

## Abstract

**Background and objective:**

There are limited data on airway clearance treatment (ACT) practices. This study aimed to: 1) assess the feasibility of collecting online surveys on ACTs from patients and physiotherapists and linking the patient survey data to outcome data in the Bronch-UK/EMBARC Registry; 2) assess the association between ACT practices and outcome data; and 3) ascertain the factors affecting physiotherapist ACT practices.

**Methods:**

Survey methodology was used to collect data from patients with bronchiectasis and physiotherapists in Northern Ireland. Associations between patient survey data and linked Bronch-UK/EMBARC Registry patient outcome data were explored.

**Results:**

It was feasible to conduct an online survey with patients with bronchiectasis and link the data to the Bronch-UK/EMBARC Registry. 13% of patients did not perform ACTs. ACTs were used more often by patients who were symptomatic/had more severe disease compared to those with milder symptoms/disease. Patients used ACTs when they were symptomatic rather than as a preventative management strategy. Physiotherapists generally followed the bronchiectasis guidelines, using the stepwise approach to management.

**Conclusion:**

Our survey provided information about the feasibility of linking online survey and patient registry data. This study provides up-to-date information on ACT practice throughout the course of the disease trajectory as well as insight into the implementation of bronchiectasis guidelines by physiotherapists. Future work should explore how to optimise ACT data collection to maximise the use of real-world ACT data in bronchiectasis research and inform priority ACT research questions.

## Abstract 2

**This study provides up-to-date information on ACT practice and implementation of bronchiectasis guidelines by physiotherapists. Future work should explore how to optimise ACT data collection to maximise use of real-world ACT data in bronchiectasis research.** https://bit.ly/3AX90hQ

## Introduction

It is recognised that airway clearance treatments (ACTs) in the form of pharmacological and non-pharmacological treatments are central to facilitate early management of bronchiectasis and have increasing importance over the disease trajectory [1]. ACTs aim to facilitate sputum expectoration, to improve ventilation and reduce cough and breathlessness. In the long term, ACTs reduce further airway damage through limiting the vicious cycle of bacterial colonisation, thus reducing inflammation, number of exacerbations and hospital admissions and improving health-related quality of life (HRQoL) [2–5]. Published guidelines recommend that people with bronchiectasis should be made aware of the different ACTs available to them, techniques should be as independent as possible and preference and adherence should be taken into account when choosing the technique [2–5]. Experts advocate a stepwise approach to treatment appropriate to the stage and severity of disease, underpinned by clinical judgement [1] with a personalisation of ACT based on established physiological principles [6].

Given the limitations of traditional research methodologies in ACT research to date (a limited number of clinical trials, most of which are single treatment studies), exploration of the relationships and patterns in real-world longitudinal data may generate important evidence and provide direction on future ACT research.

The Bronchiectasis Observational Cohort and Biobank UK (Bronch-UK) and the European Multicentre Bronchiectasis Audit and Research Collaboration (EMBARC) registries collect baseline and follow-up data (including some basic ACT data) on patients with bronchiectasis throughout the UK and Europe. At the time of our study initiation, there were limited surveys on ACT practices [7, 8] and UK ACT practices have not been explored for 20 years [8].

The aims of the current study were to: 1) assess the feasibility of collecting online survey data relating to ACTs from patients and physiotherapists and linking the patient survey data to their outcome data in the Bronch-UK/EMBARC Registry; 2) assess the association between the patients’ reported ACT practices and their outcome data; and 3) ascertain the factors affecting physiotherapists’ decision making related to their patients’ ACT practices.

## Methods

### Participants

Patients from each of the participating sites in Northern Ireland (NI) (Belfast City Hospital, Antrim Area Hospital, Craigavon Hospital and Altnagelvin Hospital) with prior agreement to be re-contacted for future studies were identified from the Bronch-UK/EMBARC Registry. Only patients on the Bronch-UK/EMBARC Registry were eligible. All patients had a clinical history consistent with bronchiectasis and computed tomography demonstrating bronchiectasis. Patients with bronchiectasis due to known cystic fibrosis were excluded. Eligibility criteria for the Bronch-UK/EMBARC Registry are described in full elsewhere [9, 10].

### Physiotherapists

Physiotherapists who were currently treating people with bronchiectasis were identified by a senior physiotherapist at each NI hospital providing respiratory care to people with bronchiectasis.

### Survey design and content

The research team conducted patient and physiotherapist focus groups, semi-structured interviews [11] and obtained feedback from patient representatives from the European Lung Foundation and Belfast City Hospital to inform the content and format of the final patient and physiotherapist surveys.

The online survey was delivered using Survey Monkey. Invitations featured a hyperlink and quick access code, distributed *via* post or e-mail. Agreeable patients completed the consent form and survey online. Patients could call the study team to complete the survey over the phone or to complete a paper version and post back. Reminders were sent at 1, 3 and 6 months. Consenting patients were contacted 9–12 months after completion of the first survey to complete a second survey. To facilitate linkage of the survey data to the registry data, patients entered their Bronch-UK/EMBARC Registry unique study ID to retain anonymity and confidentiality of the patient's personal data.

Registry data accessed for this study included demographics, lung function, total exacerbations in the preceding year, Modified Medical Research Council dyspnoea scale (mMRC), sputum, *Pseudomonas aeruginosa* status, bronchiectasis severity index (BSI) and HRQoL.

The purpose of the online patient surveys was to ask patients about their experience using ACTs and their current practices when they felt well and unwell (increase in symptoms or unwell with a chest infection). In the patient information sheet, ACTs were defined as chest physiotherapy/exercises that help to remove mucus from the lungs and cough it out.

Invitations to the physiotherapist survey featured a hyperlink, and quick access codes were distributed *via* e-mail. Reminders were sent at 1, 3 and 6 months.

The purpose of the online physiotherapist survey was to explore current ACT practice and respiratory physiotherapy services for bronchiectasis in NI.

### Statistical analysis

Data were analysed descriptively using RStudio 4.1.0. Appropriate descriptive statistics were used. The categorised variables for frequency and duration of ACT were combined to achieve a dose of ACT. High dose included patients who performed ACT at a high duration (>10 min) by high frequency (daily); medium dose included patients who performed ACT at a high duration (>10 min) by low frequency (monthly), high duration (>10 min) by medium frequency (1–3 times weekly), and low duration (<10 min) by high frequency (daily). Low dose included patients who performed ACT at a low duration (<10 min) by low frequency (monthly) and low duration (<10 min) by medium frequency (1–3 times weekly). Additionally, data were categorised and analysed according to use of non-pharmacological ACT adjunct (*i.e.* using a device) *versus* ACTs non-adjuncts (*i.e.* not using a device) status, as well as the use of mucoactives (pharmacological ACTs) *versus* no mucoactives. Associations between patient-reported ACT usage and clinical outcomes (main comparisons made when registry data were available within 6 months) were assessed using appropriate parametric and non-parametric statistics. Simple logistic and multiple regression were used to assess the effects of outcomes on the probability of a patient using ACTs and using mucoactives.

### Ethics

Full ethical approval (REC Reference: 19/SC/0528) and research governance permissions from each participating Health and Social Care (HSC) Trust were in place at the respective sites. Informed consent was provided by all participants.

## Results

### Feasibility of data collection using online surveys

The first patient survey was distributed to 398 patients with bronchiectasis from the Bronch-UK/EMBARC Registry, and the survey was completed by 205 (52%) individuals between October 2020 and October 2021. Some questions were only completed by patients who were taught ACTs (n=188) or who performed ACTs (n=177).The second patient survey was distributed to all 205 patients who completed the first survey and was completed by 96 (47%) individuals. The physiotherapist survey was distributed to 100 physiotherapists reported to provide a bronchiectasis service and was completed by 48 (48%) individuals between January 2020 and January 2021. The number of responses varied for each question within the surveys; therefore the total number of responses to each question is included in this article and missing responses were excluded.

The majority of patients (55%, 113 out of 205) self-administered the first survey online, 33% (67 out of 205) completed it over the phone with the researcher and 12% (25 out of 205) completed a paper version. The majority of patients (94%, 90 out of 96) self-administered the second survey online, whilst 6% (6 out of 96) completed it over the phone with the researcher. All physiotherapists (n=48) self-administered independently online.

### Feasibility of linking survey data to patient outcome data in the registry

Patient outcome data from the Bronch-UK/EMBARC Registry were available for 176 out of 205 (86%) patients who completed the first survey. Only 53% (108 out of 205) of patients had a registry review visit within 6 months of completing the first survey, and these data are presented as the main analysis. Their demographics and outcome data are presented in supplementary table S1, and there were no differences between the cohort with registry data and those without. Only 28% (30 out of 108) who completed the second survey had a registry review within 6 months of the second survey.

### Patients’ ACT practices

#### Use of ACTs

At the time of survey completion, the majority of patients performed ACTs (86%, 177 out of 205); however, 14% (28 out of 205) did not perform ACTs.

The linked outcome data from the Bronch-UK/EMBARC Registry for the 108 patients (93 performed ACTs, 15 did not perform ACTs) who completed the survey who had a Registry review within 6 months are presented in table 1. Demographics and lung function parameters were similar in patients who did and did not perform ACTs. Whilst limited by small numbers in subgroups, patients who performed ACTs were more likely to have had a higher total number of exacerbations in the preceding year (p=0.03) and had a worse quality of life (QoL) according to QoL – bronchiectasis (QoL-B) treatment burden domain (p=0.04), compared to those who did not perform ACTs. There were no differences in the other outcome data. Simple logistic regression modelling did not demonstrate significance in the effect of patient outcome data on the probability of using ACTs (supplementary table S2.1).

**TABLE 1:** Comparison of demographics/outcome data of patients who did *versus* did not perform airway clearance techniques (ACTs)

**Variables from Bronch-UK/EMBARC Registry**	**Reported in the survey: performed ACT**				
	**Yes**		**No**		
	**n**		**n**		**p-value (95% CI)**
**Demographics**					
Age years	93	67 (57–73)	15	70 (60–80)	0.1 (−13.00–1.00)
Sex, female	93	57 (61)	15	8 (53)	0.76
Smoking status (Yes)	93	2 (2)	15	2 (2)	0.1
BMI kg·m^−2^	29	25 (22–29)	3	29 (26–30)	0.37 (−8.60–5.35)
**Lung function**					
FEV_1_ L	42	2±1	4	2±1	0.42 (−1.35–0.70)
FEV_1_ % predicted	31	88 (56–102)	3	81 (71–97)	0.78 (−62.84–52.37)
Mild (>80%)		16 (52)		2 (67)	1
Moderate–severe (≤80%)		15 (48)		1 (33)	
**Pulmonary exacerbations**					
Total exacerbations in the preceding year	93	2 (0–3)	15	1 (0–2)	0.03 (0.0000042–2.00)
No exacerbations		24 (26)		6 (40)	0.04
1–2 exacerbations		31 (33)		8 (53)	
≥3 exacerbations		38 (41)		1 (7)	
**mMRC**					
Overall	93	1 (1–3)	15	1 (1–2)	0.59 (−0.00002–1.00)
Grade 0–1		13 (14)		2 (13)	1
Grade 2–4		80 (86)		13 (87)	
**Sputum**					
Produces daily sputum (Yes)	93	82 (88)	15	11 (73)	0.25
Sputum colour	81		11		
Mucoid		43 (53)		9 (82)	0.16
Mucopurulent		23 (28)		2 (18)	
Purulent/severely purulent		15 (19)		0 (0)	
Sputum volume mL	82	18 (6–40)	11	12 (6–38)	0.75 (−6.84–12.76)
Mild (<10 mL)		25 (30)		4 (36)	0.96
Moderate–severe (≥10 mL)		57 (70)		7 (64)	
***Pseudomonas aeruginosa* (Yes)**	93	10 (11)	15	0 (0)	0.39
**BSI**	92	8 (5–12)	15	6 (4–12)	0.48 (−2.00–3.00)
**QoL-B domains**					
Physical functioning	86	40 (20–67)	10	37 (18–77)	0.92 (−26.60–20.00)
Role functioning	87	53 (33–70)	11	67 (30–83)	0.38 (−26.70–13.30)
Vitality	86	44 (22–56)	11	44 (22–61)	0.89 (−11.10–22.20)
Emotional functioning	86	75 (50–92)	11	75 (62–92)	0.49 (−25.00–8.30)
Social functioning	87	44 (25–67)	11	67 (46–75)	0.07 (−33.30–0.00001)
Treatment burden	80	56 (44–78)	8	78 (64–92)	0.04 (−33.40– −0.00005)
Health perceptions	87	42 (25–58)	11	50 (21–54)	0.83 (−16.60–16.70)
Respiratory symptoms	87	59 (41–74)	11	59 (51–80)	0.36 (−22.20–7.40)

Data represent patients who had linked outcome data from the Bronch-UK/EMBARC Registry within 6 months of survey completion (n=108 out of 205). Data are presented as n (%), mean±sd or median (25–75% interquartile range). BMI: body mass index; FEV_1_: forced expiratory volume in 1 s; mMRC: Modified Medical Research Council dyspnoea scale; BSI: bronchiectasis severity index; QoL-B: quality of life – bronchiectasis.

The linked outcome data from the Bronch-UK/EMBARC Registry for the 176 patients showed similar results and are presented in supplementary table S3.

In the survey, the majority of patients (68%, 140 out of 205) reported being first taught about ACTs by a physiotherapist at an outpatient appointment at a hospital clinic; 8% (17 out of 205) said they had never been taught ACTs (supplementary figure S1).

Most patients ranked seeing a physiotherapist who was a specialist in bronchiectasis as important for their first ACT visit (supplementary figure S2). The most important factors considered by patients to be included in their first visit with a physiotherapist were: being taught how to perform ACTs, receiving a chest assessment and receiving information on the importance of ACTs (supplementary figures S2 and S3). Of patients who had been taught ACTs, 73 out of 184 (40%) reported that they had not been followed up for their ACT by a physiotherapist.

#### Types of ACTs

**FIGURE 1:**
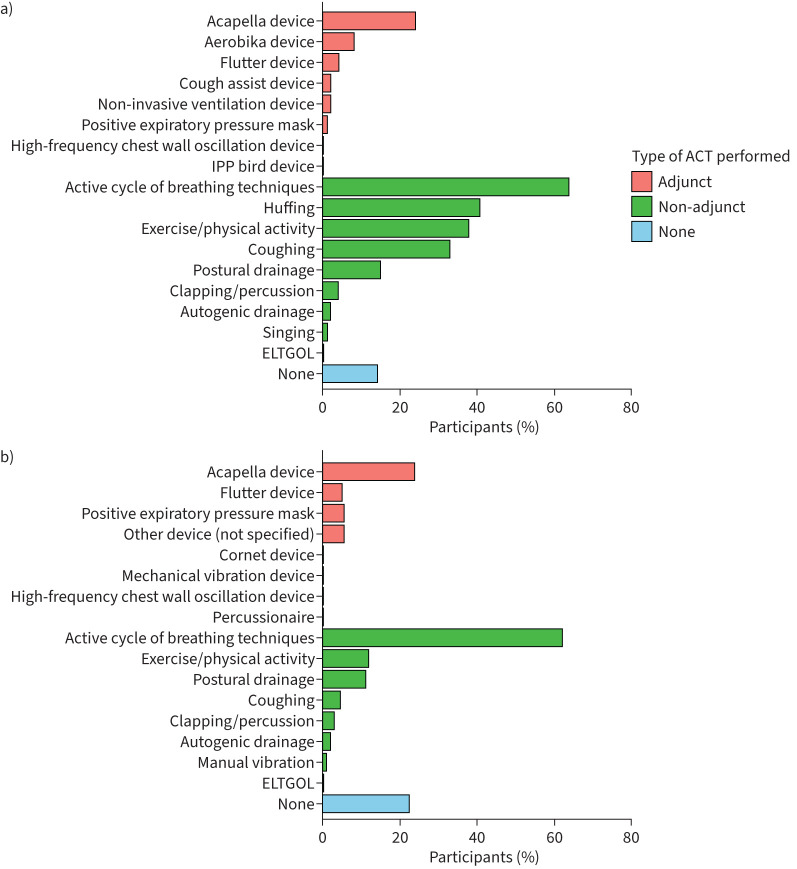
a) Types of airway clearance treatments (ACTs) used by patients in the survey (n=201). b) Types of ACTs used by patients in the Bronch-UK Registry.

In the survey, Active Cycle of Breathing Techniques (ACBT) (64%, 129 out of 201) was the most commonly reported ACT, followed by huffing (41%, 83 out of 201) and exercise and/or physical activity (38%, 76 out of 201). Of the few patients who reported using adjunct ACTs, the Acapella (24%, 49 out of 201), Aerobika (8%, 16 out of 201) and Flutter (4%, 9 out of 201) devices were most common (figure 1a). The specific use of ACTs in NI is similar to use throughout the UK (Bronch-UK registry data, figure 1b) [12].

The linked outcome data from the Bronch-UK/EMBARC Registry for the 108 patients (43 used non-adjuncts alone, 50 used adjuncts, either alone or in combination with non-adjuncts) who had a Registry review within 6 months are presented in table 2. Demographics and lung function parameters were similar in patients who used non-adjuncts and adjuncts. There were no differences in the other outcome data.

**TABLE 2:** Comparison of demographics/outcome data of patients who reported using non-adjuncts alone *versus* adjuncts (either alone or in combination with non-adjuncts)

**Variables from Bronch-UK/EMBARC Registry**	**Reported in the survey: type of ACT performed**				
	**Non-adjuncts**		**Adjuncts**		
	**n**		**n**		**p-value (95% CI)**
**Demographics**					
Age years	43	69 (59–74)	50	64 (55–72)	0.06 (−11.00–0.00006)
Sex, female	43	21 (49)	50	36 (72)	0.04
Smoking status (Yes)	43	0 (0)	50	2 (2)	0.01
BMI kg·m^−2^	12	26 (24–30)	17	23 (22–27)	0.22 (−5.39–1.49)
**Lung function**					
FEV_1_ L	16	2±1	26	2±1	0.94 (−0.47–0.51)
FEV_1_ % predicted	13	95 (62–110)	18	73 (54–97)	0.25 (−40.09–11.07)
Mild (> 80%)		8 (62)		8 (44)	0.57
Moderate–severe (≤80%)		5 (38)		10 (56)	
**Pulmonary exacerbations**					
Total exacerbations in the preceding year	43	1 (0–3)	50	2 (1–4)	0.15 (−0.00003–1.00)
No exacerbations		14 (33)		10 (20)	0.24
1–2 exacerbations		15 (35)		16 (32)	
≥3 exacerbations		14 (33)		24 (48)	
**mMRC**					
Overall	43	1 (1–2)	50	1 (1–3)	0.71 (−0.00007–1.00)
Grade 0–1		8 (19)		5 (10)	0.37
Grade 2–4		35 (81)		45 (90)	
**Sputum**					
Produces daily sputum (Yes)	43	37 (86)	50	45 (90)	0.79
Sputum colour	37		45		
Mucoid		22 (59)		22 (49)	0.24
Mucopurulent		7 (19)		16 (36)	
Purulent/severely purulent		8 (22)		7 (16)	
Sputum volume mL	37	18 (6–30)	45	30 (6–40)	0.31 (−0.92–12.24)
Mild (<10 mL)		11 (30)		14 (31)	1
Moderate-severe (≥10 mL)		26 (70)		31 (69)	
***Pseudomonas aeruginosa* (Yes)**	43	4 (9)	50	6 (12)	0.93
**BSI**	43	7 (5–10)	49	8 (5–12)	0.22 (−1.00–3.00)
**QoL-B domains**					
Physical functioning	39	40 (20–70)	47	40 (20–60)	0.78 (−13.40–13.30)
Role functioning	40	53 (32–68)	47	47 (33–67)	0.81 (−13.30–6.70)
Vitality	39	44 (33–56)	47	44 (22–56)	0.61 (−11.10–11.10)
Emotional functioning	39	75 (54–96)	47	75 (50–83)	0.44 (−16.70–8.30)
Social functioning	40	46 (24–69)	47	44 (25–67)	0.75 (−16.60–8.40)
Treatment burden	34	67 (47–86)	46	56 (36–67)	0.15 (−22.20–0.00003)
Health perceptions	40	42 (25–58)	47	42 (17–58)	0.42 (−16.60–8.30)
Respiratory symptoms	40	62 (43–78)	47	56 (39–74)	0.42 (−14.80–7.40)

Data represent patients who had linked outcome data from the Bronch-UK/EMBARC Registry within 6 months of survey completion (n=108 out of 205^#^). Data are presented as n (%), mean±sd or median (25–75% interquartile range). ACT: airway clearance treatment; BMI: body mass index; FEV_1_: forced expiratory volume in 1 s; mMRC: Modified Medical Research Council dyspnoea scale; BSI: bronchiectasis severity index; QoL-B: quality of life – bronchiectasis. ^#^: n=15 out of 108 excluded as they reported they did not perform ACTs.

The linked outcome data from the Bronch-UK/EMBARC Registry for the 176 patients are presented in supplementary table S4.

#### ACT dose

**FIGURE 2:**
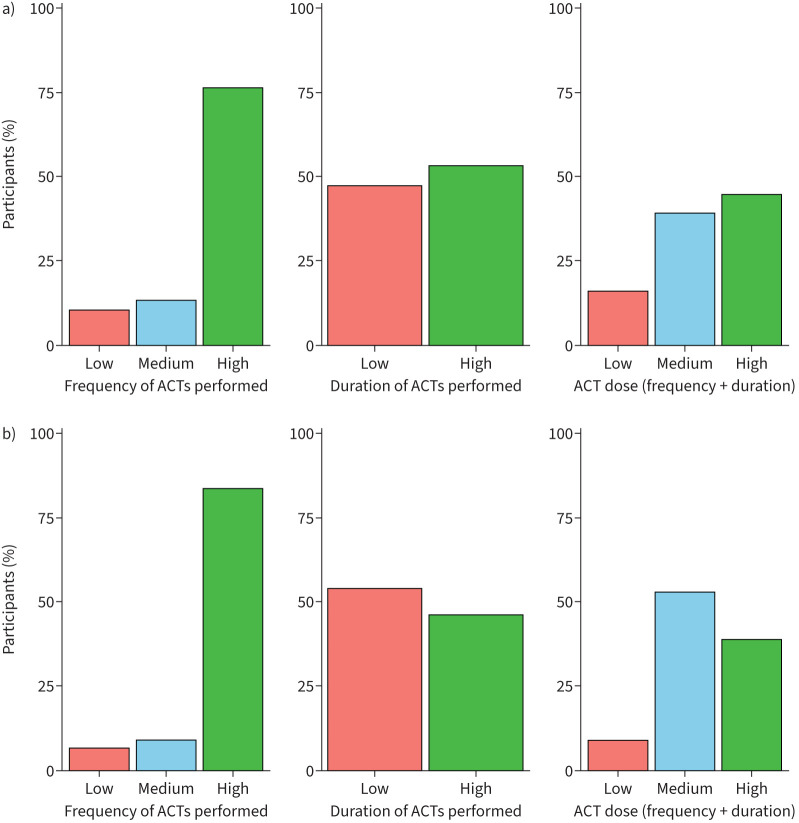
a) Frequency, duration and dose of airway clearance treatments (ACTs) performed by patients when well (n=177). b) Frequency, duration and dose of ACTs performed by patients when unwell (n=177).

In the survey, 145 out of 173 (84%) patients performed either a medium or high dose of ACTs when well. In the survey, a higher number of patients (91%, 158 out of 173) performed a medium or high dose of ACTs when unwell compared to when well (figure 2). When unwell, the majority of patients (79%, 136 out of 173) did not change the type of ACT they performed, 12% (21 out of 173) changed their ACT type, 4% (7 out of 173) increased their ACT dose and 5% (9 out of 173) increased pharmacological support (supplementary figure S4). When unwell, patients reported that ACT frequency and duration depended on clearing their chest (28%, 48 out of 173), how much sputum they had (25%, 43 out of 173) and their physiotherapist's instructions (24%, 41 out of 173) (supplementary figure S5).

The majority of patients found that ACTs helped to prevent chest infections (75%, 131 out of 174) and alleviate sticky (68%, 119 out of 174) or excessive sputum (68%, 118 out of 174 (supplementary figure S6).

The linked outcome data from the Bronch-UK/EMBARC Registry for the 108 patients (45 performed a high dose, 32 reported a medium dose and seven reported a low dose of ACTs when well) who had a Registry review within 6 months are shown in supplementary table S5. Demographics and lung function parameters were similar in patients who performed a high, medium and low dose of ACTs. Compared to those who performed a low dose of ACTs, patients who performed a high or medium dose of ACT had worse QoL than patients who performed a low dose according to QoL-B treatment burden (p=0.04) and respiratory symptoms (p=0.04) domains. There were no differences in the other outcome data.

The linked outcome data from the Bronch-UK/EMBARC Registry for the 176 patients show similar results and are presented in supplementary table S6.

#### Mucoactives and other medications to help ACTs

In the survey, patients reported taking bronchodilators (45%, 89 out of 199), hypertonic saline (35%, 69 out of 199) and carbocisteine (32%, 63 out of 199) (supplementary figure S7). Fewer patients reported taking other medications including isotonic saline, DNase, mannitol, antibiotics and steroids. The majority of patients report taking their medications before their ACTs, with the exception of carbocisteine; 54% (34 out of 63) of patients said they did not time their carbocisteine around their ACTs (supplementary figure S8). 23% (45 out of 199) of patients said they did not take any medications to help with their ACTs.

The linked outcome data from the Bronch-UK/EMBARC Registry for the 108 patients (53 used mucoactives and 55 patients did not use mucoactives) who had a Registry review within 6 months are presented in table 3. Lung function parameters and exacerbation rates were similar in patients who did and did not use mucoactives. Compared to those who did not use mucoactives, patients who used mucoactives: were younger (p=0.004); had a lower mMRC grading (p=0.006); had a higher BSI score (p=0.03) and worse QoL across several QoL-B domains: physical functioning (p=0.02), vitality (p=0.001), treatment burden (p=0.0001) and respiratory symptoms (p=0.004). There were no differences in the other outcome data.

**TABLE 3:** Comparison of demographics/outcome data of patients who reported using or not using mucoactive medications

**Variables from Bronch-UK/EMBARC Registry**	**Reported in the survey: uses mucoactive medications**				
	**Yes**		**No**		
	n		n		**p-value (95% CI)**
**Demographics**					
Age years	53	63 (54–71)	55	70 (60–77)	0.004 (−12.00– −2.00)
Sex, female	53	37 (70)	55	28 (51)	0.08
Smoking status (Yes)	53	3 (6)	55	1 (2)	0.44
BMI kg·m^−2^	18	24 (22–32)	14	26 (23–27)	0.78 (−3.80–4.74)
**Lung function**					
FEV_1_ L	29	2±1	17	2±1	0.52 (−0.55–0.28)
FEV_1_ % predicted	21	76±30	13	87±33	0.33 (−34.26–12.00)
Mild (>80%)		11 (52)		7 (54)	1
Moderate–severe (≤80%)		10 (48)		6 (46)	
**Pulmonary exacerbations**					
Total exacerbations in the preceding year	53	2 (0–5)	55	1 (0–3)	0.14 (−0.000004–1.00)
No exacerbations		14 (26)		16 (29)	0.5
1–2 exacerbations		17 (32)		22 (40)	
≥3 exacerbations		22 (42)		17 (31)	
**mMRC**					
Overall	53	1 (1–2)	55	2 (1–3)	0.006 (0.00003–1.00)
Grade 0–1		4 (8)		11 (20)	0.11
Grade 2–4		49 (92)		44 (80)	
**Sputum**					
Produces daily sputum (Yes)	53	46 (87)		47 (85)	1
Sputum colour	46		49		
Mucoid		25 (54)		28 (60)	0.67
Mucopurulent		12 (26)		13 (28)	
Purulent/severely purulent		9 (20)		6 (13)	
Sputum volume mL	46	20 (6–4)	47	2 (1–3)	0.67 (−4.08–10.00)
Mild (<10 mL)		15 (33)		14 (30)	0.94
Moderate–severe (≥10 mL)		31 (67)		33 (70)	
***Pseudomonas aeruginosa* (Yes)**	53	7 (13)	55	3 (5)	0.29
**BSI**	52	9 (5–12)	55	6 (4–10)	0.03 (0.00001–3.00)
**QoL-B domains**					
Physical functioning		33 (15–53)		47 (27–80)	0.02 (−26.70– −0.00004)
Role functioning		47 (22–65)		53 (40–80)	0.14 (−20.00–0.00007)
Vitality		33 (22–53)		56 (33–67)	0.001 (−22.30– −11.1)
Emotional functioning		67 (50–83)		75 (58–92)	0.08 (−16.70–0.00003)
Social functioning		42 (18–67)		50 (33–67)	0.14 (−22.30–0.00003)
Treatment burden		56 (33–67)		78 (56–89)	0.0001 (−33.30– −11.10)
Health perceptions		33 (25–50)		50 (25–58)	0.09 (−16.70–0.00001)
Respiratory symptoms		52 (38–69)		67 (44–82)	0.004 (−22.20– −3.70)

Data represent patients who had linked outcome data from the Bronch-UK/EMBARC Registry within 6 months of survey completion (n=108 out of 205). Data are presented as n (%), mean±sd or median (25–75% interquartile range). BMI: body mass index; FEV_1_: forced expiratory volume in 1 s; mMRC: Modified Medical Research Council dyspnoea scale; BSI: bronchiectasis severity index; QoL-B: quality of life – bronchiectasis.

The linked outcome data from the Bronch-UK/EMBARC Registry for the 176 patients showed similar results and are presented in supplementary table S7.

In simple logistic regression modelling of the probability of using mucoactives, the effect of age, mMRC, BSI and QoL-B domains (physical functioning, vitality, respiratory symptoms and treatment burden) were significant. The probability of using a mucoactive diminished by: 4% for every year increase in age; 2% for every 1-point increase in QoL-B physical functioning; 3% for every 1-point increase in QoL-B vitality; 3% for every 1-point increase in QoL-B respiratory symptoms; 4% for every 1-point increase in QoL-B treatment burden. The probability of using a mucoactive increased by: 62% for every 1-unit increase in mMRC and 12% for every 1-unit increase in BSI (supplementary table S2.1). In multiple logistic regression modelling, when controlling for all these factors, only age and QoL-B treatment burden remained significant (supplementary table S2.2.).

### Follow-up survey (9–12 months later)

In the second survey, the majority (78%, 70 out of 90) of patients reported performing the same type of ACT with no change in ACT frequency (72%, 65 out of 90), duration (68%, 61 out of 90) and dose (54%, 49 out of 90). The majority (80%, 72 out of 90) also used the same mucoactive medications.

### Airway clearance practices: the physiotherapist's perspective

Physiotherapists ranked patient symptoms as the most important factor influencing choice of ACTs and ranked performing a physiotherapy chest assessment and providing patients with information on ACTs as the most important factors involved in a first visit for patients with bronchiectasis (table 4). Patient access to a respiratory physiotherapist, regardless of whether they specialised in bronchiectasis, was ranked as most important for patient follow-up (table 4). Patient symptoms and disease stability were considered the most important factors in prioritising patients for follow-up visits (table 4).

**TABLE 4:** Factors influencing physiotherapists’ decision making regarding airway clearance treatments (ACTs) for patients with bronchiectasis

**Factors**	**Mean rank of importance**
**1. Influencing choice of ACTs**	
** **My knowledge and experience of using the ACT	4.8**^#^**
** **Availability and access to the ACT	5.8**^#^**
** **Comorbidities of the person with bronchiectasis	6**^#^**
** **Understanding and competence of the person with bronchiectasis	3.8**^#^**
** **Time allocated to the individual's appointment	8.2**^#^**
** **Preferences of the person with bronchiectasis for certain ACTs	4.4**^#^**
** **Symptoms of the person with bronchiectasis	2.4**^#^**
** **Disease stability of the person with bronchiectasis	5.1**^#^**
** **Disease severity of the person with bronchiectasis	5.1**^#^**
** **Local tariffs or prescriptions/funding available for equipment	9.5**^#^**
**2. Content for a first visit for ACTs**	
** **Providing information about ACTs and why they are important	2.3^¶^
** **Performing a physiotherapy chest assessment	1.7^¶^
** **Teaching an ACT	3.1^¶^
** **Setting a personal action plan for ACTs with the patient	4.6^¶^
** **Having enough time for the first appointment	4.7^¶^
** **Providing information about other physiotherapy treatments in addition to ACTs	5.5^¶^
** **Providing information about support groups	7.7^¶^
** **Providing contact details and instruction on how to access a physiotherapist in the future	6.3^¶^
**3. Content for a first follow-up visit**	
** **Patient access to a physiotherapist who is specialised in bronchiectasis	2.2**^+^**
** **Patient access to a physiotherapist who works with respiratory patients who is not a specialist	2**^+^**
** **Patient visit in a dedicated bronchiectasis clinic	2.8**^+^**
** **Patient visit in a location of their choice	3**^+^**
**4. Priorities of a follow-up visit**	
** **Symptoms of the person with bronchiectasis	2^§^
** **Comorbidities of the person with bronchiectasis	4.7^§^
** **Understanding and competence of the person with bronchiectasis with their ACT	3^§^
** **Disease stability of the person with bronchiectasis	2.2^§^
** **Disease severity of the person with bronchiectasis	3.3^§^
** **Local tariffs or prescriptions/funding available for equipment	5.9^§^

**^#^**: 1=most important, 10=least important; ^¶^: 1=most important, 8=least important; ^+^: 1=most important, 4=least important; ^§^: 1=most important, 6=least important.

## Discussion

In the current study, we used an online survey to collect data on ACT practices from the patients’ and physiotherapists’ perspective and linked this data to patient outcome data in the Bronch-UK/EMBARC Registry; this provided an in-depth insight into ACT practices in bronchiectasis.

The overall patient and physiotherapist response rate aligned with mean response rates reported in a recent systematic review of surveys [13]. In the current study, although over half (55%, 113 out of 205) of patients responded online independently, a large proportion (45%, 92 out of 205) requested to complete by other means (including interview mode and *via* post), indicating that this population still requires support for completing an online survey. Similarly, Meyer *et al*. [13] reported higher response rates for in-person and postal surveys (77% and 68% respectively) compared to online surveys (59%).

In the British Thoracic Society (BTS) guidelines [5] and BTS Quality Standards [14], it is recommended that ACTs should be taught to all patients with bronchiectasis; however a small number of patients in the current survey reported that they had never been taught ACTs. Data from the EMBARC registry [15] found that only 48.3% of patients performed ACTs regularly with the primary reason for not doing ACTs being that it was not required in the opinion of the clinicians (67.9%). Data from the US Bronchiectasis Registry [16] found that more than one-half of patients (58%) who used ACTs at baseline did not report the use of ACTs at 1-year follow-up. In contrast, in the current survey, 78% of patients reported performing the same type of ACT at 9–12 months follow-up. One possible reason for the difference may be that the majority of hospitals in NI have designated respiratory physiotherapy services.

Published guidelines [2, 4, 5] and BTS Quality Standards [14] provide guidance on assessment, treatment and follow-up on ACT. This survey showed the specific use of ACTs in NI is similar to use throughout the UK (Bronch-UK Registry data). Most patients reported using ACBT, huffing and exercise and/or physical activity; previous studies in bronchiectasis have reported similar findings [7, 8, 15, 17]. Positive expiratory pressure and oscillating positive expiratory pressure were used less frequently than ACBT in the UK and are also used less than in other countries, *e.g.* Australia and New Zealand [7]. This is not surprising as some ACTs have geographical dominance; they tend to be closely related to their country of origin and/or the undergraduate training in the country [16, 18, 19].

The subgroup and regression analyses in this study provide a novel insight into ACT practice, with data showing that those who use ACT and indeed those who used a higher dose of ACT were sicker patients. Those patients who used mucoactives were younger and more likely to have a bigger treatment burden. This may reflect the clinical experience that some older patients can be challenged by polypharmacy/treatment burdens for their “other” conditions, and mucoactives may be less likely to be considered as a result. These results also reflect that patients may be using ACT symptomatically rather than prophylactically and may also reflect instructions and subsequently the preferences of the physiotherapist managing the patients.

In the current study, the results indicate that physiotherapists are generally following the bronchiectasis guidelines and using the stepwise approach to management: patients are using manual ACTs when they are experiencing milder symptoms, and as their symptoms become more severe, adjunct type ACTs and mucoactives are also being used. Similar results have been shown in the US Bronchiectasis Registry [16].

Patients and physiotherapists both highlighted that it was important that patients were seen by a specialised physiotherapist for ACTs, and most patients reported being first taught ACTs by a physiotherapist; this aligns with what is recommended in the guidelines and quality standards. Despite this, the latest National Bronchiectasis Audit (2017) [20] highlighted there was suboptimal assessment of adult patients by a respiratory physiotherapist. Based on our survey results, we suggest the quality statement should expand recommendations to fully capture adherence to guidelines.

The latest Bronchiectasis Audit (2017) [20] collected high-level data on physiotherapy input, *i.e.* whether patients were seen by a respiratory physiotherapist and whether they had a self-management plan however, it did not collect granular detail on ACTs, *i.e.* ACT type, frequency, duration, dose and how these change when well/ unwell. We propose that more detail on ACTs is included in future audits to assess the adherence to quality standards. This level of detail would optimise the real-world data collected, to enable review and facilitate longitudinal studies of the linkage of ACT and patient clinical status.

### Study limitations

This study was limited to patients enrolled on the EMBARC/Bronch-UK Registry consenting to the survey. We were unable to explore changes in ACTs with patient outcome data over time due to large timeframes between each database review and completion of the surveys. Better coordination of registry and future survey initiatives will result in more complete and reliable longitudinal data.

### Conclusion

Our survey provided information about the feasibility of conducting an online survey in bronchiectasis patients and linking the data to a patient registry. This study provides information on ACT practice throughout the course of the disease trajectory. Future work should explore how to optimise ACT data collection to maximise the use of real-world ACT data in bronchiectasis research and inform priority ACT research questions. More in-depth information will enable fuller utilisation and exploration of real-world evidence on ACT effects.
